# A spectrum of cognitive-behavioral-movement disorders in adrenoleukodystrophy: A case series from a tertiary care centre in the eastern part of India

**DOI:** 10.5339/qmj.2024.43

**Published:** 2024-09-23

**Authors:** Debaleena Mukherjee, Peyalee Sarkar, Alak Pandit, Biman Kanti Ray, Gautam Das, Souvik Dubey

**Affiliations:** 1 Bangur Institute of Neurosciences, Institute of Post Graduate Medical Education & Research, SSKM Hospital, Kolkata, India *Email: drsouvik79@gmail.com

**Keywords:** Adrenoleukodystrophy, cognition, stereotypies, behavioral dysfunction, visual dysfunction

## Abstract

**Background:**

Adrenoleukodystrophy (ALD) is an intriguing disease with a heterogeneous clinico-radiological profile. Behavioral and cognitive impairments are often the initial and predominant manifestations, yet their patterns are frequently overlooked. This study aims to elaborate on the patterns of cognitive dysfunction, behavioral changes, and movement disorders in ALD to facilitate its earlier diagnosis.

**Methods:**

In this case series, 12 cases of ALD were assessed and evaluated for cognitive, behavioral, and movement abnormalities to identify patterns of involvement.

**Results:**

All patients were male, with an age range of 5–46 years. 75% presented with cerebral ALD (CALD), and 25% had an adrenomyeloneuropathy phenotype. Cognitive dysfunction, behavioral changes, and seizures were observed in 75%, 66.7%, and 33.3% of ALD patients. An initial posterior to anterior pattern of progression of cognitive impairment dominated by higher-order visual dysfunction and language regression was observed in 66.7% of CALD patients, while a frontal pattern was noted in 22.2% of CALD patients. While cognitive impairment typically indicated dysfunction of occipito-parieto-temporal networks, behavioral changes predominantly suggested dysfunctional fronto-temporal-subcortical connections. A novel observation was the occurrence of tics and stereotypies in 33.3% of ALD patients.

**Conclusion:**

This study describes the patterns of cognitive, behavioral, and movement abnormalities in ALD and highlights the contributory role of dysfunctional white matter networks. Cognitive patterns predominantly reflect a posterior-to-anterior gradient of impairment of white matter connections, while behavioral markers indicate involvement of fronto-temporal-subcortical networks. Adding to this spectrum, the occurrence of tics and stereotypies is a unique observation in ALD.

## 1. Introduction

Leukodystrophies are genetically determined leukoencephalopathies resulting from varied involvement of central nervous system white matter structural components and molecular processes.^1^ Adrenoleukodystrophy (ALD) is an X-linked metabolic disorder resulting from ABCD1 gene mutations. It has an estimated incidence of approximately 1 in 17,000 newborns. The many presentations of this clinico-radiologically diverse entity include cerebral ALD (childhood, adolescent, and adult onset), adrenomyeloneuropathy (AMN), Addison’s disease, olivo-ponto-cerebellar variants, milder spectrums in women, as well as asymptomatic phenotypes.^2-4^ Cognitive impairment and behavioral abnormalities are often the initial and predominant manifestations, yet their patterns in ALD have been inadequately explored in existing literature and practically less recognized.^2,5–7^ This has time and again resulted in delayed diagnosis coupled with multiple different specialty initial visits and referrals.^2^ Furthermore, movement disorders in ALD, including tics and stereotypies, are an intriguing association that has not been elucidated earlier. ALD requires biochemical or molecular studies for final confirmation and has a poor prognostic outcome, with current therapeutic options mostly limited to the early stages of the disease. Earlier detection by recognizing the clinical patterns can bridge the gap between symptom onset, diagnosis, and therapy, thereby substantially alleviating the mental and financial burden. This study delves deeper into and elaborates on the clinical patterns of cognitive-behavioral-movement disorders in ALD in different age groups, along with their motor and endocrinological associations and radiological profiles.

## 2. Methods

**Figure 1.**
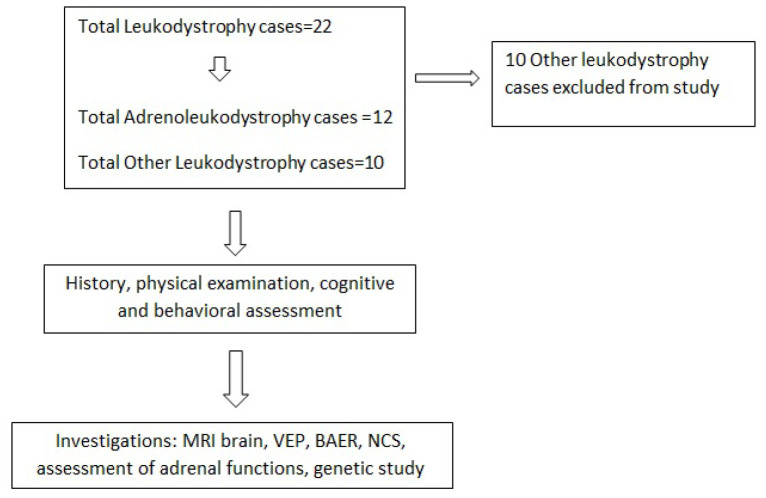
Methodology.

This descriptive study is a case series from a single tertiary care institute for neurology in the eastern part of India, spanning over 2 years (December 2020 to January 2023). Among a total of 22 cases of leukodystrophies registered at the institute, 12 were cases of ALD, which were confirmed by genetic studies and/or classical radiological features. All consecutive cases of ALD were included and underwent thorough history-taking, examination, and laboratory investigations as follows (Figure 1).

### 2.1. Cognitive assessment

The patients underwent age-adjusted assessments of cognitive domains as per the DSM-5 including attention, executive functions, language, learning and memory, perceptual-motor, social cognition, praxis, and calculations, as follows:^8^

1) Attention: Basic attention during conversation (eye contact, ability to keep track of conversation, alerting to sound and visual stimuli), digit span test (forward and backward), and A vigil test.
2) Executive: Similarities, lexical fluency, motor series (Luria), conflicting instructions, go-no-go, and prehension behavior as part of the frontal assessment battery.^9^
3) Praxis: Pantomine (transitive and intransitive), gesture imitation, and real object use.
4) Language: Spontaneous speech, simple comprehension, complex comprehension, syntactic comprehension, fluency, repetition, naming, reading, and writing.
5) Learning and memory: Word list memory task, free recall, delayed word list memory task, and visual memory.
6) Calculation: Simple and complex calculations.
7) Visuoperceptual:
  - For apperceptive agnosia: copying, matching objects.
  - For perceptual categorization defects: fragmented letter test, silhouettes, unusual view test, and foreshortened matches.
  - For associative agnosia: object description, sorting into categories, matching functionally similar but visually dissimilar objects, and differentiating real from unreal objects.
  - Prosopagnosia: informal facial recognition test.
  - Cerebral color vision abnormalities: Ishihara chart, color plate naming, pointing to color, matching color, conceptual color naming, and color painting.
8) Visuospatial:
  - Simultanagnosia: Dorsal: Modified letter cancellation test and large global letter made of smaller local letters; Ventral: description of a composite picture.
  - Optic ataxia.
  - Oculomotor apraxia.
  - Atereopsis/Akinetopsia: Historical correlates.
9) Global cognitive assessment: Clock drawing.

### 2.2. Behavioral assessment

This was done using the Neuropsychiatric Inventory Questionnaire.^10^

All patients were critically analyzed for patterns of cognitive and behavioral impairment and their relation to other neuroaxis involvement.

### 2.3. Motor assessment

Pyramidal (spasticity), extrapyramidal (dystonia and other movement disorders), cerebellar features, and peripheral neuropathy were noted.

### 2.4. Investigations

These included 3Tesla magnetic resonance imaging (MRI) of the brain [Verio 3T Siemens, Magnetom], electroencephalography, visual evoked potentials (VEP), brainstem auditory evoked response (BAER) studies, nerve conduction studies, and assessment of adrenal functions (serum electrolytes, cortisol-adrenocorticotrophic hormone [ACTH] levels). Genetic confirmation was done using clinical exome sequencing studies, which allow the detection of variability in the protein-coding region of any gene rather than targeting a few selective genes.

Written informed consent was duly obtained from all patients or guardians, and the study commenced after obtaining institutional ethical clearance.

## 3. Results

The study incorporated a total of 12 subjects with ALD, which constituted greater than half (54.5%) of the total leukodystrophy cases evaluated. All patients (100%) were male. The age at presentation was 20.92 ± 13.91 years (range 5-46 years), with the majority presenting in the first to the second decade of life. Cerebral ALD was the predominant phenotype (75%), followed by AMN (25%). The clinical features and investigations are detailed as follows (Tables 1 and 2).

### 3.1. Clinical profile

***3.1.1. Cerebral ALD (CALD)***

The mean age of our CALD population at the time of presentation was 19.56 ±13.58 years. The age range at the time of onset of CALD was 5–44.5 years and at the time of diagnosis, it was 5–46 years. Approximately two-thirds were childhood and adolescent onset CALD. Cognitive impairment, behavioral changes, and seizures were the major manifestations observed in 75%, 66.7%, and 33.3% of all ALD patients. The initial pattern of cognitive impairment reflected a dysfunction of posterior predominant white matter networks in 66.7%, anterior in 22.2%, and global in 11.1% of CALD patients. A posterior to anterior pattern of progression of cognitive impairment was typically observed in the majority, with higher-order visual dysfunction (HOVD) and partial Gerstmann-like presentation (language regression and dyscalculia) being the initial features, followed by impairment of memory, executive functions, and attention. An anterior predominant cognitive impairment with attention and executive dysfunction dominating the picture was observed in two cases. Overall memory, visuospatial, executive, language, and attention domains were affected in 88.9%, 77.8%, 77.8%, 66.7%, and 55.6% of CALD patients, respectively. Praxis could not be formally assessed in nearly half (55.6%) due to involvement of other domains, while it was normal in the rest. Importantly, while the cognitive impairment was posteriorly predominant, the behavioral abnormalities suggested major involvement of the fronto-temporal-subcortical networks. Among behavioral changes, inappropriate laughter, agitation/anger, and apathy were most common (25%, 16.7%, and 16.7% of ALD patients), followed by hyperorality, obsessive-compulsive disorder (OCD), autism spectrum disorder (ASD), impulse control disorder (ICD), paranoid delusions, auditory, and visual hallucinations (8.3% each). A striking observation, highlighting the cognitive-behavioral-motor continuum, was the occurrence of tics and stereotypies in one-third of ALD patients (33.3%) (Video 1). It is noteworthy that both patients with anterior-predominant dysfunction presented with stereotypical head movements. Cervical tics and stereotypy of the face, neck, and upper extremities were also noted in two cases of posterior predominant dysfunction. Spasticity was observed in more than two-thirds of the subjects with CALD. Generalized dystonia along with cerebellopathy were observed in one.

***3.1.2. Adrenomyeloneuropathy (AMN)***

The age of presentation of subjects with the AMN phenotype was 25 ± 17.09 years. The age range at the time of onset of AMN was 9–39 years, and at the time of diagnosis, it was 9–43 years. Generalized dystonia was observed in two subjects, one of whom also demonstrated cerebellopathy (Video 2). Peripheral neuropathy was not observed in any of our cases. Notably, one of the patients demonstrated apathy without any cognitive impairment.

Overall, spasticity, dystonia, and cerebellopathy were observed in 83.3%, 25%, and 16.7% of all ALD patients, respectively.

The duration of symptoms prior to diagnosis ranged from 2 months to 5 years in cases of CALD and 6 months to 4 years in cases of AMN. We noted that every patient in our study had an average of four different specialty visits prior to a final diagnosis of ALD.

### 3.2. Investigations

***3.2.1. Radiological correlates***

**Figure 2.**
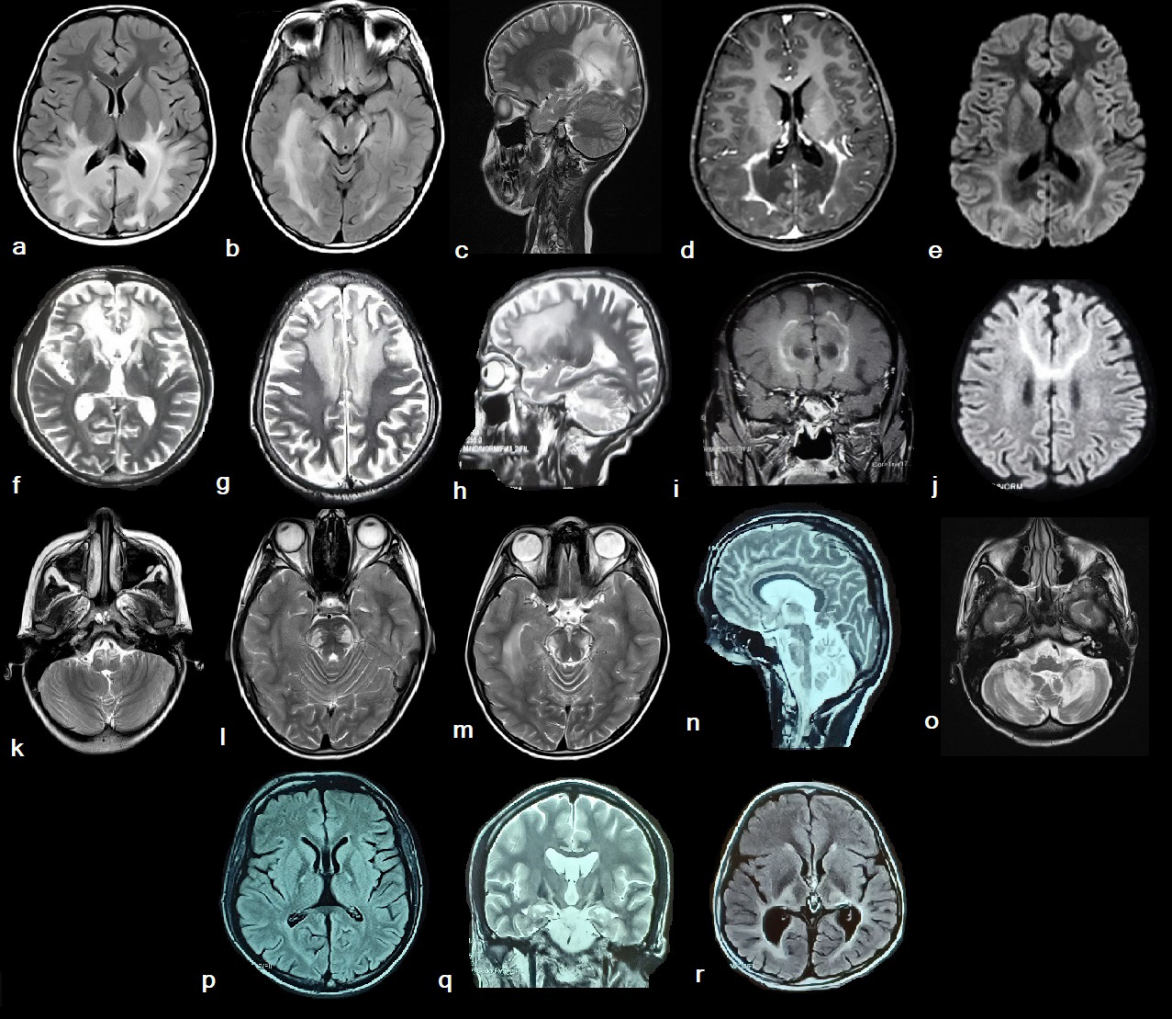
Radiological profile of patients with ALD on magnetic resonance imaging of the brain including T2, T2 Flair, contrast, and DWI sequences. Row 1: (a–e) Parieto-occipital pattern of ALD with involvement of parieto-occipital white matter (a, c), occipito-temporal white matter (b), and splenium of corpus callosum (a), with contrast enhancement (d), and DWI changes (e). Row 2: (f–j) Frontal pattern of ALD with involvement of frontal white matter (f, g, h), genu of corpus callosum (f), with contrast enhancement (i), and DWI changes, “stag horn appearance” (j). Row 3: (k–o) Posterior fossa involvement in ALD with hyperintensity along corticospinal tracts in medulla (k), pons (l), and midbrain (m), cerebellar atrophy (n), and cerebellar hyperintensities (o). Row 4: (p–r) White matter hyperintensity noted along bilateral corticospinal tracts (p, q) with a “wine glass appearance” on coronal view (q). Bilateral thalamic involvement in ALD (r).

All patients had abnormalities in the MRI brain, with greater than two-thirds (75%) showing typical symmetrical hyperintensities of occipito-parieto-temporal white matter and the splenium of the corpus callosum (Loes et al., pattern 1).^11^ A frontal pattern of ALD (pattern 2) was noted in 16.7%. Isolated hyperintensity along the corticospinal tract (CST) was observed in a single case (8.3%, pattern 3) who also demonstrated cerebellar atrophy. Hyperintensities of CST along with pattern 1 were noted in four cases. Overall, posterior fossa involvement was noted in 41.7%, including cerebellar atrophy, cerebellar hyperintensities, and signal changes along CST in the brainstem. Bilateral thalamic involvement was noted in 16.7%. Contrast enhancement was present in 75%. Hyperintensity along the white matter tracts on diffusion-weighted imaging (DWI) without true restriction was present in two-thirds (66.7%) of cases (Figure 2).

***3.2.2. VEP/BAER***

VEP studies showed prolonged P100 latencies in 58.3% of ALD patients. These patients presented with HOVD and normal fundoscopic examinations. BAER was abnormal in two patients who had impaired auditory discrimination. Pure-tone audiometry could not be reliably tested as the patients were inattentive.

***3.2.3. Adrenal insufficiency***

This was observed in one-third of patients, with three cases having CALD and one having AMN, without overt systemic manifestations. It was detected on the basis of normal cortisol and elevated ACTH levels.

***3.2.4. Genetic study***

Clinical exome sequencing was done as a confirmatory test for all, except three patients due to financial limitations. Hemizygous missense variations in exon 1 of the ABCD1 gene were observed in three patients, while missense variations in exons 7 and 9, nonsense variation in exon 10, and a 3′ splice site variation in intron 7 were present in single cases each. Two cases had base-pair deletions in exons 1 and 10.

## 4. Discussion

Cerebral ALD (CALD) was the most common presentation in our study, followed by AMN. Dysfunction in cognitive and behavioral domains dominated the clinical picture. The noteworthy observation of tics and stereotypies in our subjects further expanded the existing clinical spectrum. With currently available therapies limited to hematopoietic stem cell transplantation and gene therapy applicable only in the early stages of disease, ALD warrants an early diagnosis. Further importance lies in the identification and management of adrenal insufficiency, which may occur isolated or as a part of the disease spectrum.^4^ Thus, early recognition of the cognitive-behavioral-movement disorder patterns in ALD can be instrumental in minimizing diagnostic delays and unwanted referrals.

### 4.1. Cognitive patterns

In our study, CALD majorly presented in the 1st –2nd decades. Similar to previous authors, childhood-onset CALD was the most common age of presentation.^2,4^ Delayed developmental milestones, neuroregression, and cognitive dysfunction are known features of ALD, yet their patterns are often missed. Cases being tagged as sequelae of hypoxic ischemic encephalopathy, especially when a history of birth asphyxia is present, are an oversimplification, as observed in some of our cases. Thus, in addition to delayed milestones, identifying regressing milestones in children in their developmental phase is vital, where recently acquired and less consolidated functions are the first to be lost. Declining scholastic performance was a common, broad complaint reported by the parents of ALD-affected children. A meticulous dissection revealed several plausible causes, including higher-order visual dysfunction, auditory agnosia, language regression, dyscalculia, inattention, and impairment of memory.

*Higher-order visual dysfunction* (HOVD), resulting from involvement of the occipito-parietal and occipito-temporal white matter connections, was the initial and predominant complaint in greater than two-thirds of cases. Some previous authors have also highlighted the greater impairment of visual-motor, visuospatial skills, and visual memory in ALD.^2,12^ Overall, we observed a greater involvement of the “where pathways” compared to the “what pathways.” HOVD and the consequent lack of “visual attention” often led to children being perceived as inattentive or mischievous, consequently being stamped with a misdiagnosis of attention deficit hyperactivity disorder (ADHD).^2,4^ It also commonly masqueraded as an anterior visual pathway abnormality, resulting in multiple unyielding ophthalmology visits. An important clue in the majority was that visual problems were reported by caregivers rather than the patients themselves, as is common in cases of anterior visual pathway dysfunctions. Adding to this confusion were abnormal VEP studies (prolonged P100 latencies) with normal fundoscopic examinations, resulting in extensive and futile search for an anterior visual pathway disease. This was observed in greater than half, possibly indicating a functional disruption and extension of white matter involvement from optic radiations to the optic tracts. Notably, optic atrophy was not observed in any of our patients. *Auditory dysfunction* was less frequently observed in comparison to visual. Word deafness has been described previously.^2^ An impairment of frequency-specific auditory discrimination was noted in two of our patients. Involvement of the middle part of the superior temporal gyrus and its white matter connections is likely contributory. *Language regression* was observed in two-thirds of CALD patients, and it often closely accompanied visual impairment. An initial difficulty in reading and a regression of writing skills to scribbling were quickly followed by a reduction in verbal and gestural expressions and a gross impairment of basic comprehension. This possibly resulted from a dysfunction of the perisylvian white matter pathways of the dominant hemisphere involving the occipito-temporo-parietal connections from the occipital lobe to the visual word formation area and its connections with semantics, the lexical retrieval system, and the angular gyrus. In two patients, the difficulty in reading and writing was more attributable to letter agnosia and simultanagnosia. Partial Gerstmann syndrome-like presentation with language regression and acalculia was also noted. In contrast to the initial presentation with HOVD and language regression, a predominant initial impairment of *attention and executive functions* resulting from disruption of fronto-parietal and frontal-subcortical connections was observed in only two patients. In others, these functions were lost over the course of disease evolution. “Forgetfulness” was often reported by caregivers; however, on detailed analysis, it was mostly attributable to dysfunction of other cognitive domains rather than memory. Although overall memory was the most commonly affected domain, impairment of recent episodic memory was present as one of the initial manifestations only in two cases, while in others it developed with disease progression. This likely resulted from disruption of the white matter connections in the papez circuit and fronto-temporal networks.

### 4.2. Behavioral patterns

Cognitive and behavioral changes share a dynamic relationship. Lack of recognition of behavioral patterns has commonly resulted in delayed and inappropriate diagnoses, including ADHD, intellectual disability, schizophrenia, mania, and bipolar disorder.^5,7,13^ We observed that while cognitive impairment typically had a pattern of progression from posterior to anterior white matter connections, behavioral changes largely reflected greater impairment of the fronto-temporal-subcortical networks.

*Inappropriate laughter and smiling* were observed in three subjects. The discordant emotion reflected a pseudobulbar affect likely resulting from loss of inhibitory control of the orbitomedial frontal cortex via corticobulbar tracts on the ventral pontomedullary center.^14^ Importantly, while one of these cases had a frontal pattern with respect to cognitive impairment and radiological profile, the two others were posterior predominant. *Agitation, anger, and aggression* were other commonly reported complaints, the onset of which usually accompanied or followed HOVD. One may deduce that the loss of visual sensory cues leads to a heightened state of arousal and stress reactivity, confusion, and agitation. In addition to sensory deprivation, a dysfunctional fronto-temporo-limbic circuit may also be responsible.^15,16^ These patients radiologically demonstrated occipito-parieto-temporal white matter hyperintensities. In contrast to agitation, *apathy* was also noted, which is often difficult to assess in the background of language regression, dysarthria, and motor disability. It was observed in two subjects, with eventual progression to an akinetic mute state in one who also had a frontal pattern of cognitive and radiological involvement. Abnormality of the frontal-subcortical connections (salience network) involving the medial prefrontal cortex and anterior cingulate gyrus is implicated.^17^ Interestingly, apathy was also observed in a patient with the AMN phenotype without any cognitive impairment but showing cerebral involvement radiologically (Loes pattern 3), perhaps hinting at the possibility of a future clinical progression to CALD.

*Hyperorality and biting tendencies* were observed in a single case in the background of HOVD, resembling a partial Kluver–Bucy syndrome. Radiologically, occipito-parieto-temporal white matter hyperintensities were present. The plausible explanation is that the loss of a major sensory modality like vision leads to the re-emergence of phylogenetically primitive senses, including hyperorality and olfaction, which serve as survival techniques like food searching and defensive behaviors. Norman Geschwind also emphasized a disconnection syndrome interrupting visual stimuli in the limbic circuit as the basis for Kluver–Bucy syndrome.^18,19^

*Obsessive-compulsive* features along with cervical tics in early childhood were observed in one case that later developed HOVD and recent memory impairment in late childhood. The behavioral features, along with tics, can stem from the involvement of the cortico-striato-thalamo-cortical (CSTC) circuits, involving the orbitofrontal cortex, anterior cingulate cortex, caudate, and thalamus. Notably, the patient had parieto-occipital white matter hyperintensities with sparing of the anterior anatomic correlates for OCD and tics.^20^ *Impulsivity* evolving into multiple drug addictions in a background of autism in childhood was the initial presentation in one patient, once again harping on the involvement of CSTC circuits including the limbic, associative, and sensori-motor loops.^21^ He eventually developed *auditory and visual hallucinations* in a background of multi-domain cognitive impairment, including higher-order visual and auditory dysfunction, in adulthood. The hallucinations possibly resulted from faulty processing of multimodal, higher-order sensory inputs.^22^ Radiologically, Loes pattern 1 was noted. *Paranoid delusions* were observed in a single patient who had, over time developed apathy followed by an akinetic mute state with stereotypical head movements, once again implicating the frontal-subcortical connections involving the dorsolateral and ventromedial prefrontal cortex, ventral striatum, hippocampus, substantia nigra, and mesolimbic pathways.^23^ Clinical findings correlated radiologically with a frontal pattern.

From labile affect to broader autistic phenocopies to neurotic and psychotic spectrums, our study highlights the pivotal role of dysfunction of white matter connections involving CSTC loops, fronto-limbic circuits, and fronto-parietal-subcortical networks. Subtle changes in personality and behavior can be the earliest clues of disease onset. Two patients, though majorly symptomatic in late childhood and adulthood, demonstrated pre-existing behavioral problems in the form of obsessive compulsive features, autistic traits, and impulse control disorder. Whether these monosymptomatic behavioral abnormalities, which preceded the typically known manifestations of ALD, indicate an expanded spectrum warrants future studies. While CALD is known to have a rapidly progressive course, adolescent and adult onsets can have a slower disease evolution as compared to childhood onset, as was also observed in our study.^2^ Adolescent and adult-onset CALD may have long-standing behavioral abnormalities with decompensation and a full-blown spectrum at a later age.^24^ Identifying these personality traits and behavioral changes early in childhood can serve as potential disease harbingers. It is noteworthy that even though the behavioral changes majorly suggested involvement of fronto-temporal- subcortical networks, the MRI brain revealed only parieto-occipito-temporal white matter hyperintensities in several of the cases. This indicates that behavioral markers result from functional disruption of white matter networks, which may precede the appearance of anatomical and radiological correlates.

### 4.3. Seizures

Seizures were observed in nearly one-third of patients, typically generalized tonic clonic seizure (GTCS) in two and focal seizures with impaired awareness (FIAS) in another two.

### 4.4. Motor abnormalities

***4.4.1. Spasticity***

It was the most common motor manifestation, affecting all patients with the AMN phenotype and greater than two-thirds of CALD subjects.

***4.4.2. Tics and stereotypies***

A novel observation in 33.3% of ALD patients was the presence of tics and stereotypical movements. In two cases, stereotypical head movements were one of the initial manifestations, along with cognitive and behavioral impairment suggestive of frontal-subcortical dysfunction. A third patient developed stereotypical movements in a cranio-cervico-brachial distribution during the course of disease evolution, starting with auditory agnosia and HOVD. Another patient had cervical tics in the background of OCD and higher-order visual and recent memory impairment. This highlights the cognitive-behavior-movement disorder interface resulting from abnormal functioning of the cortico-striato-pallido-thalamo-cortical loop. Such movement disorders are uncommon in leukodystrophies, and to the best of our knowledge, this association has not been described earlier in ALD. It necessitates the need to exclude ALD as a differential in such future cases.^25^

***4.4.3. Dystonia***

It was observed in 25% of the study population, in the background of spasticity in two AMN patients and in one CALD case. While the basal ganglia is primarily implicated in the pathophysiology of dystonia, disruption of the white matter network model for sensory-motor integration via striato-thalamo-cortical connections may be a plausible explanation for our patients. Notably, there was no demonstrable radiological basal ganglia abnormality except for thalamic changes in two cases of AMN, underscoring the role of the thalamus in dystonia.^26-28^

***4.4.4. Cerebellopathy***

Cerebellar involvement, either isolated or as part of the spectrum, has been reported earlier by previous authors. Cerebellopathy along with spastic dystonia were observed in two patients (16.7%), one without cognitive impairment and the other with florid behavioral and cognitive dysfunction. This also correlated with marked cerebellar atrophy and hyperintense cerebellar signal changes on imaging. This emphasizes the need to consider ALD as a differential for adult-onset ataxias.^29,30^

### 4.5. Radiological aspects

Loes et al. described five main radiological patterns of ALD, including pattern 1: parieto-occipital white matter, pattern 2: frontal white matter, pattern 3: corticospinal tract, pattern 4: cerebellar white matter, and pattern 5: concomitant parieto-occipital and frontal white matter.^11^ In our study, pattern 1 was most common, followed by patterns 2 and 3. All patients with an initial posterior predominant cognitive impairment had Loes pattern 1, and those with frontal predominant decline had Loes pattern 2. Interestingly, two patients with AMN demonstrated Loes pattern 1, while pattern 3 was observed in the third. Patients with a predominant AMN phenotype also demonstrating cerebral signal changes without initial cognitive impairment may suggest that radiological dysfunction can precede clinical cognitive decline and a possible future evolution into CALD.^2^ Contrast enhancement and DWI changes without true restriction similar to active demyelination were also commonly noted, signifying disease activity. The DWI changes in frontal ALD resembled a “stag horn appearance.” The white matter hyperintensities along the corticospinal tract resembled the typical “wine glass appearance” commonly described in motor neuron disease and even in Krabbes, but not previously described in ALD.^31,32^

### 4.6. Genetics

Abnormalities of exon 1 were most commonly encountered in four patients in the form of missense variations and base pair deletions. Similar to that of previous authors, there was no demonstrable pheno-genotypic correlation in our study.^4,33^

## 5. Study Limitations

The subjects were recruited from a single tertiary care center for neurological diseases, thus creating the possibility of a selection bias. In view of the small sample size, which limits statistical analysis, descriptive data has been provided in this study.

## 6. Conclusion

Cognitive patterns predominantly have a posterior to anterior gradient of dysfunction of white matter networks, manifesting most commonly as higher-order visual dysfunction and language regression. Behavioral markers are often early and subtle, predominantly reflecting dysfunction of the fronto-temporal-subcortical networks. It can precede radiological correlates, possibly suggesting a functional disruption. The novel observation of tics and stereotypies highlights the intriguing cognitive-behavioral-motor continuum in ALD. Thus, identification of these clinical patterns along with their radiological profiles can facilitate earlier diagnosis and therapeutic initiation in ALD in the future.

## Table of Abbreviations

**Table:** 

**Abbreviations**	**Term**
ACTH	Adrenocorticotrophic hormone
ADHD	Attention deficit hyperactivity disorder
ALD	Adrenoleukodystrophy
AMN	Adrenomyeloneuropathy
ASD	Autism spectrum disorder
BAER	Brainstem auditory evoked response
CALD	Cerebral Adrenoleukodystrophy
CNS	Central nervous system
CST	Corticospinal tract
CSTC	Cortico-striato-thalamo-cortical
DSM	Diagnostic and statistical manual of mental disorders
DWI	Diffusion-weighted imaging
EEG	Electroencephalogram
FIAS	Focal seizures with impaired awareness
HOVD	Higher order visual dysfunction
ICD	Impulse control disorder
MRI	Magnetic resonance imaging
NCS	Nerve conduction studies
NPI-Q	Neuropsychiatric Inventory Questionnaire
OCD	Obsessive compulsive disorder
VEP	Visual evoked potentials

## Conflict of Interest Statement

There is nothing to declare as competing interest for any of the authors, including the corresponding author. I had full access to all of the data in this study, and I take complete responsibility for the integrity of the data and the accuracy of the data analysis.

## Ethics Approval and Consent to Participate

All subjects and patient guardians provided written informed consent for aggregate and anonymous reporting of the data arising from their clinical assessments. The study was approved by the Institutional Ethics Committee of IPGME&R and SSKM Hospital, in full accordance with international standards for the ethical use of human subjects in research. IPGME&R/IEC/2020/568. Written informed consent was obtained from all patients/guardians for this study and its publication.

## Data Availability Statement

The authors confirm that all the data generated or analyzed during the study are included within the article and/or its supplementary materials.

## Declaration of Originality, Authorship

This manuscript is based on original work and has not been published, in whole or in part, in any print or electronic media or is under consideration for publication in any print or electronic media other than as an abstract of conference proceedings.

## Authors’ Contributions

All persons listed as authors in the manuscript have made substantial contributions, so as to take public responsibility for it, in the production of this manuscript, as detailed below: DM conceptualized and wrote the manuscript; PS, AP, BR, and GD edited the manuscript; and SD conceptualized and edited the manuscript. All authors read and approved the final manuscript. No person who contributed substantially to the production of this manuscript has been excluded from authorship. As the corresponding author of this manuscript, I have recorded the statements of each author regarding any competing interests as per the checklist provided.

## Supplementary Material

Video 1. Stereotypy in ALD.

CASE 1. Stereotypical movements involving the face, neck, and upper extremities and inappropriate laughter in a 10-year-old male. He presented with higher-order auditory and visual dysfunction and language regression, followed by global impairment and seizures, along with a parieto-occipital pattern of radiological involvement.

CASE 2. Stereotypical movements of the head (no-no type) in a 30-year-old male who presented with impairment of attention, executive functions, memory along with emotional lability, and a frontal pattern radiologically.

CASE 3. Stereotypical movements of the head (no-no type) in a 46-year-old male who presented with inattentiveness, paranoid delusions, wandering, and apathy, progressing to an akinetic mute state with a frontal pattern radiologically.

Video 2. A 23-year-old male with generalized dystonia, appendicular ataxia, and spastic-ataxic gait.

https://zenodo.org/records/13778016?token=eyJhbGciOiJIUzUxMiJ9.eyJpZCI6IjdhODg3ZWJkLWFiOTEt NGU3OC1iOTE0LTViMGZiNWE0YWI1NSIsImRhdGEiOnt9LCJyYW5kb20iOiI0YThiMzk0YWZlZWE2N2Q0YTAwMTNkYWVmYjJiZjI5ZiJ9.OKRrZv02XGWyDOxQMt23WHeY8UjHUEbAMtBWlXeizcWmQ_YEDsU70eUF_3sxO6jaI9k9Fx3FkIeof0oWDr0aVQ

## Figures and Tables

**Table 1.** Clinical profile and investigations of patients with adrenoleukodystrophy.

	**Age (yrs)**	**Gender**	**Disease duration**	**No. of initial visits**	**Cognitive impairment pattern**	**Behavioral pattern**	**Seizure**	**Motor**	**Adrenal insufficiency**	**MRI Brain**	**VEP**	**Genetic study**
1	5	M	3 mon	3	Higher order visual + language regression → global impairment	Aggression, Biting, Hyperorality	GTCS	None	Yes	Loes pattern 1, Brainstem WMH+, CE +, DWI change +.	Abn	Hemizygous nonsense variation in exon10 of the ABCD1gene
2	9	M	6 mon	5	-	-	-	Spastic dystonia	No	Loes pattern 1, Bilateral thalamic involvement +, No CE.	N	Hemizygous missense variation in exon7 of ABCD 1 gene
3	10	M	2 mon	3	Auditory agnosia → Higher order visual dysfunction + Language regression → Global impairment	Inappropriate laughter	GTCS	Spasticity, Stereotypy of face and upper extremities.	Yes	Loes pattern 1, Brainstem WMH +, CE +. DWI change +.	Abn VEP & BAER	Hemizygous 3 base pair deletion in exon 1 of the ABCD1 gene
4	13	M	6 mon	5	Recent episodic memory + higher order visual dysfunction	OCD	FIAS	Spasticity, Cervical tics	No	Loes pattern 1, CE +, DWI change +.	Abn	Hemizygous 4 base pair deletion in exon 10 of the ABCD1gene
5	18	M	5 yrs	5	Higher order visual dysfunction +,language regression + dyscalculia + executive dysfunction	-	FIAS	-	No	Loes pattern 1, No CE	Abn	-
6	23	M	2 yrs	6	-	Apathy	-	Spasticity, Generalized dystonia, Pan-cerebellopathy	Yes	Loes pattern 3, Thalamic involvement+, Cerebellar atrophy +, No CE.	N	Hemizygous missense variation in exon1 of ABCD gene
7	32	M	5 yrs	5	Multidomain cognitive decline including auditory and visual agnosia	Autism spectrum disorder, Impulse control disorder, Addictions, Auditory/visual hallucination		Spasticity, Generalized dystonia, Pan-cerebellopathy	Yes	Loes pattern 1, Brainstem WMH+, Cerebellar hyperintensities +, CE+, DWI change +	Abn VEP & BAER	Hemizygous 3′ splice site variation in intron 7 of the ABCD1 gene
8	43	M	4 yrs	3	-	-	-	Spasticity	No	Loes pattern 1, Brainstem WMH + CE +, DWI change +	N	Hemizygous missense variation in exon 9 of ABCD1 gene
9	30	M	1.5 yrs	4	Attention, Recent memory, executive impairment	Emotional lability (pseudobulbar affect)	-	Spasticity, Stereotypical head movements	No	Loes pattern 2, CE +	N	Hemizygous missense variation in exon 1 of ABCD gene
10	14	M	8 mon	4	Higher order visual dysfunction + language+ recent memory + executive	Anger, foul language, inappropriate smiling	-	Spasticity	No	Loes pattern 1, CE +, DWI change +	Abn	-
11	8	M	6 mon	3	Higher order visual dysfunction	-	-	Spasticity	No	Loes pattern 1, CE+, DWI change +	Abn	-
12	46	M	1.5 yrs	5	Attention → Multidomain cognitive decline	Irritability, paranoid delusions, Wandering, Apathy → akinetic mute	-	Stereotypy of head, Spasticity	No	Loes pattern 1, CE +, DWI change +	N	Hemizygous missense variation in exon 1 of ABCD gene

M, male; Yrs, years; mon, months; GTCS, generalized tonic-clonic seizure; FIAS, focal seizure with impaired awareness; +, present; CE, contrast enhancement; DWI, diffusion-weighted imaging; WMH, white matter hyperintensity; VEP, visual evoked potentials; BAER, brainstem auditory evoked response; Abn, abnormal; N, normal.

**Table 2.** Distribution of cognitive dysfunctions, behavioral patterns, movement abnormalities, radiological characteristics, and genetic associations among ALD cases.

**Cognitive impairment**		**Behavioral changes**		**Movement disorders**		**Radiology**		**Genetic study**
Posterior predominant	66.7%	Inappropriate laughter/smiling	25%	Tics and stereotypies	33.3%	Parieto-occipito-temporal WMH	75%	Missense variations (exons 1, 7, and 9)
Anterior predominant	22.2%	Anger/Aggression	16.7%	Dystonia	25%			
		Apathy	16.7%	Ataxia	16.7%			
Global	11.1%	Hyperorality	8.3%					
		OCD	8.3%			Frontal WMH	16.7%	Nonsense variation (exon 10)
		ASD/ICD/Addictions	8.3%					
		Paranoid delusions	8.3%					
		Auditory/Visual hallucinations	8.3%					
						WMH along CST	8.3%	3′ splice site variation (intron 7)
						Cerebellar	16.7%	Deletions (exon 1 and 10)
(Represents the initial pattern at presentation among CALD subjects)		(Represents patterns among all ALD subjects)						

OCD, obsessive-compulsive disorder; ASD, autism spectrum disorder; ICD, impulse control disorder; WMH, white matter hyperintensity; CST, corticospinal tract.
